# 20.4 MODELING THE CONTRIBUTION OF COMMON VARIANTS TO SCHIZOPHRENIA RISK

**DOI:** 10.1093/schbul/sby014.083

**Published:** 2018-04-01

**Authors:** Gabriel Hoffman, Brigham Hartley, Douglas Ruderfer, Judith Rapoport, Pamela Sklar, Kristen Brennand

**Affiliations:** 1 Icahn School of Medicine at Mount Sinai; 2 NIH

## Abstract

**Background:**

Schizophrenia (SZ) is a debilitating psychiatric disorder for which the complex genetic mechanisms underlying the disease state remain unclear. Whereas highly penetrant variants have proven well-suited to human induced pluripotent stem cell (hiPSC)-based models, the power of hiPSC-based studies to resolve the much smaller effects of common variants within the size of cohorts that can be realistically assembled remains uncertain.

**Methods:**

We reprogrammed fibroblasts from SZ patients into hiPSCs and subsequently differentiated these disorder-specific hiPSCs into neural progenitor cells (NPCs) and neurons. Our hiPSC neural cells, from controls and patients with SZ, better resemble fetal rather than adult brain tissue, indicating that hiPSC-based models may be best suited for studies of disease predisposition. At the cellular level, we have previously reported aberrant migration in SZ hiPSC NPCs, together with diminished neuronal connectivity and impaired synaptic function in SZ hiPSC neurons.

**Results:**

We identified microRNA-9 as having significantly downregulated levels and activity in a subset of SZ hiPSC-derived neural progenitor cells NPCs, a finding that was corroborated by a larger replication cohort and further validated by an independent gene-set enrichment analysis of the largest SZ genome-wide association study (GWAS) to date. Overall, this demonstrated a remarkable convergence of independent hiPSC- and genetics-based discovery approaches. In developing this larger case/control SZ hiPSC cohort of hiPSC-derived NPCs and neurons, we identified a variety of sources of variation, but by reducing the stochastic effects of the differentiation process, we observed a significant concordance with two large post mortem datasets.

**Discussion:**

We predict a growing convergence between hiPSC and post mortem studies as both approaches expand to larger cohort sizes. Meanwhile, we have been integrating CRISPR-mediated gene editing, activation and repression technologies with our hiPSC-based neural platform, in order to develop a scalable system for testing the effect of a manipulating the growing number of SZ-associated variants and genes in NPCs, neurons and astrocytes. Altogether, our objective is to understand the cell-type specific contributions of SZ risk variants to disease predisposition.

